# Liraglutide 3.0 mg and mental health: can psychiatric symptoms be associated to adherence to therapy? Insights from a clinical audit

**DOI:** 10.1007/s40519-023-01625-5

**Published:** 2023-11-28

**Authors:** Silvia Tempia Valenta, Michele Stecchi, Federica Perazza, Chiara Nuccitelli, Nicola Villanova, Loris Pironi, Anna Rita Atti, Maria Letizia Petroni

**Affiliations:** 1 Department of Biomedical and NeuroMotor Sciences, Alma Mater University of Bologna, Bologna, Italy; 2 IRCCS-Azienda Ospedaliera di Bologna Sant’Orsola-Malpighi, 40138 Bologna, Italy; 3 Department of Medical and Surgical Sciences, Alma Mater University of Bologna, 40138 Bologna, Italy

**Keywords:** GLP-1 receptor agonists, Adherence, Depression, Anxiety, Binge eating, Obesity

## Abstract

**Introduction:**

Liraglutide 3.0 mg, a glucagon-like peptide-1 (GLP-1) analogue, is a medication approved for obesity treatment. This study aimed to investigate the relationship between psychiatric symptoms, including depression, anxiety, and binge eating, and their impact on therapy adherence.

**Methods:**

A clinical audit was carried out on a cohort of 54 adults with obesity treated with liraglutide 3.0 mg. We retrospectively analyzed the connection between psychiatric symptoms assessed through the State-Trait Anxiety Inventory (STAI), Beck Depression Inventory (BDI), and Binge Eating Scale (BES). Adherence to therapy was assessed by the maximum dosage (MD) and treatment duration (TD).

**Results:**

Notably, a discontinuation rate of 59% was encountered. However, among those who continued the treatment, we observed a negative association between anxiety symptoms (STAI score) and MD, depression symptoms (BDI score) and TD, and a higher likelihood of binge eating (BES score > 17) and TD. Moreover, presence of psychiatric symptoms did not compromise drug's effectiveness in achieving weight loss, which was 4.43% (± 5.5 SD) in the whole sample and 5.3% (± 6.3 SD) in the subgroup evaluated at 12 weeks.

**Conclusion:**

We observed a high discontinuation rate in real-life clinical setting, where Liraglutide 3.0 therapy is paid out-of-pocket. While psychiatric symptoms might play a role in diminishing adherence to therapy, they do not prevent drug's effectiveness to promote weight loss. This finding underscores the potential advantages of liraglutide 3.0 mg therapy for individuals contending with obesity while simultaneously managing mental health challenges.

**Level of evidence:**

Level V, descriptive studies.

## Introduction

The World Health Organization (WHO) has defined adherence as “the extent to which a person's behavior, taking medication, following a diet, and/or executing lifestyle changes, corresponds with agreed recommendations from a health care provider” [1]. It is imperative to recognize that adherence is a multifaceted construct influenced by a confluence of patient-specific attributes and factors pertinent to the nature of the disease and its associated therapeutic interventions [1]. In contemporary medical discourse, obesity is increasingly acknowledged as a chronic, progressive, and recurrent ailment [2, 3]. An area of notable concern lies in the suboptimal attendance and adherence rates encountered in the context of obesity management, as substantiated by an array of empirical investigations [4, 5]. The emergence of innovative pharmacological interventions designed for prolonged therapeutic usage accentuates the exigency of an in-depth exploration into the determinants of adherence among individuals grappling with obesity.

The glucagon-like peptide-1 receptor agonists (GLP-1 RAs) function through the activation of GLP-1R receptors, exerting their effectiveness in regulating energy intake both peripherally and centrally [6, 7]. These agents enhance insulin secretion and induce satiety by acting on the hypothalamus [6, 7]. Initially introduced in 2005, GLP-1 RAs found their primary application in the treatment of type 2 diabetes (T2DM), demonstrating exceptional efficacy and a commendable safety profile in this domain [8–10]. The mechanisms of action shared by GLP-1 RAs encompass a spectrum of actions, including the augmentation of insulin secretion in response to glucose, suppression of glucagon release, delay in gastric emptying, mitigation of the release of orexigenic neuropeptides within the hypothalamus, and the promotion of anorexigenic neuropeptides [11, 12]. Collectively, these effects culminate in a reduction of body weight, body mass index (BMI), glycated hemoglobin A1c levels, and systolic blood pressure [11, 13].

In light of their broad efficacy, GLP-1 RAs have more recently been considered for treating obesity also in the absence of T2DM [14–16]. Liraglutide 3.0 mg, the first GLP-1 RA used in the treatment of obesity, was approved by Food and Drug Administration (FDA) in 2014 and by European Medicines Agency (EMA) in 2015 [17]. It has proved to be an effective tool, in adjunct to a hypocaloric diet and physical activity program, for achieving clinically relevant weight loss (≥ 5%) in adults with excess weight (BMI ≥ 27) who also have weight-related medical problems or obesity (BMI ≥ 30) [14, 18]. The findings suggest a generally favorable tolerability profile, even in the presence of common gastrointestinal side effects (i.e., primarily mild to moderately severe nausea) [18, 19].

Beyond their well-documented metabolic effects, GLP-1 RAs have emerged as noteworthy agents with potential implications for mental health [20–24]. A growing body of research has been dedicated to investigating the efficacy of GLP-1 RAs in alleviating symptoms of anxiety and depression, shedding light on a significant interplay between metabolic regulation and psychopathological mechanisms [20–24]. Recent studies have underscored the perturbation of homeostasis within the nexus of the nervous system, immune system, and endocrine system, coupled with disruptions in cerebral energy metabolism and the dysfunction of the gut–brain axis, as pivotal factors in the pathogenesis of depression and anxiety. Within this context, GLP-1 RAs, by virtue of their modulatory influence on immune, endocrine, and metabolic processes within the central nervous system, exhibit a proactive role in ameliorating these symptoms [20, 25].

Previous findings provide initial insights into the influence of liraglutide on mental health [20–24]. However, as of our current knowledge, the reciprocal impact of mental health conditions on long-term treatments like GLP-1RAs remains unexplored. Therefore, we conducted a clinical audit to investigate the interplay between psychiatric symptoms, encompassing depression, anxiety, and binge eating, and their potential effects on both adherence to and the effectiveness of liraglutide 3.0 mg therapy within our patient cohort under the care of a National Health Service outpatient obesity clinic.

## Methods

### Study design and participants

A clinical audit was conducted by retrospectively collecting data from electronic medical records (EMRs) of outpatients at the Unit of Clinical Nutrition and Metabolism, situated within Sant’Orsola-Malpighi Hospital in Bologna, Italy. The procedures described in this report constitute an integral part of our routine clinical practice. Prior to receiving clinical services, patients had granted their consent for data collection, in compliance with established privacy protocols. The audit and subsequent statistical analysis of the collated data were executed following a comprehensive anonymization process. It is standard procedure for all patients to be requested to complete a set of self-administered psychometric assessments, as outlined below.

We screened all adult outpatients, consecutively referring to the Unit of Clinical Nutrition and Metabolism from June 2019 to November 2022. Patients were considered eligible for inclusion in this study if they met the following criteria: (a) BMI ≥ 30 kg/m^2^ or BMI ≥ 27 mg/m^2^ with at least one treated weight-related comorbidity; (b) age ≥ 18 years at the time of assessment; (c) had received a prescription for liraglutide 3.0 mg by August 2022. The exclusion criteria encompassed individuals who (a) had not completed the psychometric assessment; (b) demonstrated non-adherence to the recommended dosing escalation schedule specified in the drug datasheet or as prescribed by the clinician; and (c) had a follow-up duration of less than 2 months.

It is noteworthy to emphasize that patient management within our center adheres to a well-defined protocol. Upon their initial visit, all patients are extended an invitation to engage in structured behavioral programs tailored to the severity of their excess weight and unhealthy eating habits. During subsequent follow-up appointments, patients receive motivational reinforcement aimed at facilitating lifestyle modifications and the sustained adherence to a healthy diet, as well as regular physical activity. The consideration of pharmaceutical intervention comes into play if the primary line of behavioral therapy fails to achieve the minimum anticipated outcome, defined as a weight reduction of at least 3% within a three-month period, or in cases where there is a notable history of unsuccessful prior attempts at weight loss.

According to the prescribing information for liraglutide 3.0 mg, the starting dose of 0.6 mg per day should be increased in weekly increments over 4 weeks to a recommended maintenance dose of 3.0 mg from the fifth week onward. Treatment should be discontinued after 12 weeks if the patient has not attained a weight reduction ≥ 5% of the starting weight [17] (Saxenda®; Novo Nordisk A/S, Copenhagen, Denmark). For this purpose, we considered the starting weight the one at the initial visit to the Centre.

During the Covid-19 pandemic, the majority of follow-up appointments were transitioned to remote modalities, utilizing video and phone calls. Consequently, the recording of patients’ weights at the 12-week mark was not feasible for all individuals, as this data was instead collected during their subsequent in-person examinations.

### Procedures and measures

For the purposes of this study, variables collected by clinicians as part of standard consultation and follow-up visits were used. The information was collected through EMRs and included socio-demographic data, recent and past medical history, current medications, the timing of follow-up visits, the dosage of liraglutide at the time of the visit, and side effects reported from visit to visit.

Patients filled in the Italian version of the State-Trait Anxiety Inventory (STAI) [26], Beck Depression Inventory (BDI) [27], and Binge Eating Scale (BES) [28]. STAI is a 20-item self-report assessing trait anxiety [26]. It categorizes individuals into four distinct groups based on their anxiety levels [26]. The categories include “Below threshold” for those with scores less than 40, “Mild” for individuals scoring between 40 and 49, “Moderate” for scores ranging from 50 to 59, and “Severe” for those with scores equal to or exceeding 60 [26].

BDI is a 21-item self-report measuring characteristic attitudes and symptoms of depression classifying individuals into four categories that correspond to different levels of depression [27]. These categories encompass “Below threshold” for those with depression scores below 10, “Mild” for scores between 10 and 19, “Moderate” for individuals scoring within the range of 20 to 29, and “Severe” for those with depression scores equal to or surpassing 30 [27].

BES is a 16-item self-report questionnaire designed to capture the behavioral, cognitive, and emotional features of objective binge eating in adults with obesity and overweight categorizing individuals into three distinct categories reflecting their level of body dissatisfaction or satisfaction with their body image [28, 29]. These categories consist of “Below threshold”, which represents low levels of body dissatisfaction, encompassing scores below 17 [28]. “Mild” covers moderate body dissatisfaction, with scores ranging from 17 to 26 [28]. Finally, “Severe” includes high levels of body dissatisfaction, with scores equal to or exceeding 27 [28]. BES scores above 17 are considered suggestive of significant binge eating (BE) [28].

To quantify adherence to liraglutide therapy, we collected data on maximum dosage (MD) and treatment duration (TD). To quantify the effectiveness of liraglutide therapy on weight loss, we collected data on weight at 12 weeks (or a closer follow-up examination). Therefore, the weight loss evaluation did not include patients with shorter follow-ups.

### Statistical analysis

Data analysis was performed using Statistical Package for Social Science for MacOS (SPSS) software, Version 27.0 (IBM Corp, Armonk, NY). Descriptive analyses were conducted by analyzing categorical variables’ frequencies (N) and percentages (%). Bivariate Pearson's correlations and linear regression analyses were used to investigate potential associations between psychiatric symptoms (i.e., STAI, BDI, and BES scores, BE expressed dichotomously) and adherence indicators (i.e., MD and TD). We utilized bivariate Pearson's correlations and Student's T-test to investigate potential association between psychiatric symptoms, psychoactive therapy and the percentage of weight loss. All the analyses were two-sided with α = 0.05.

## Results

### Sample selection and socio-demographic results

**Fig. 1:**
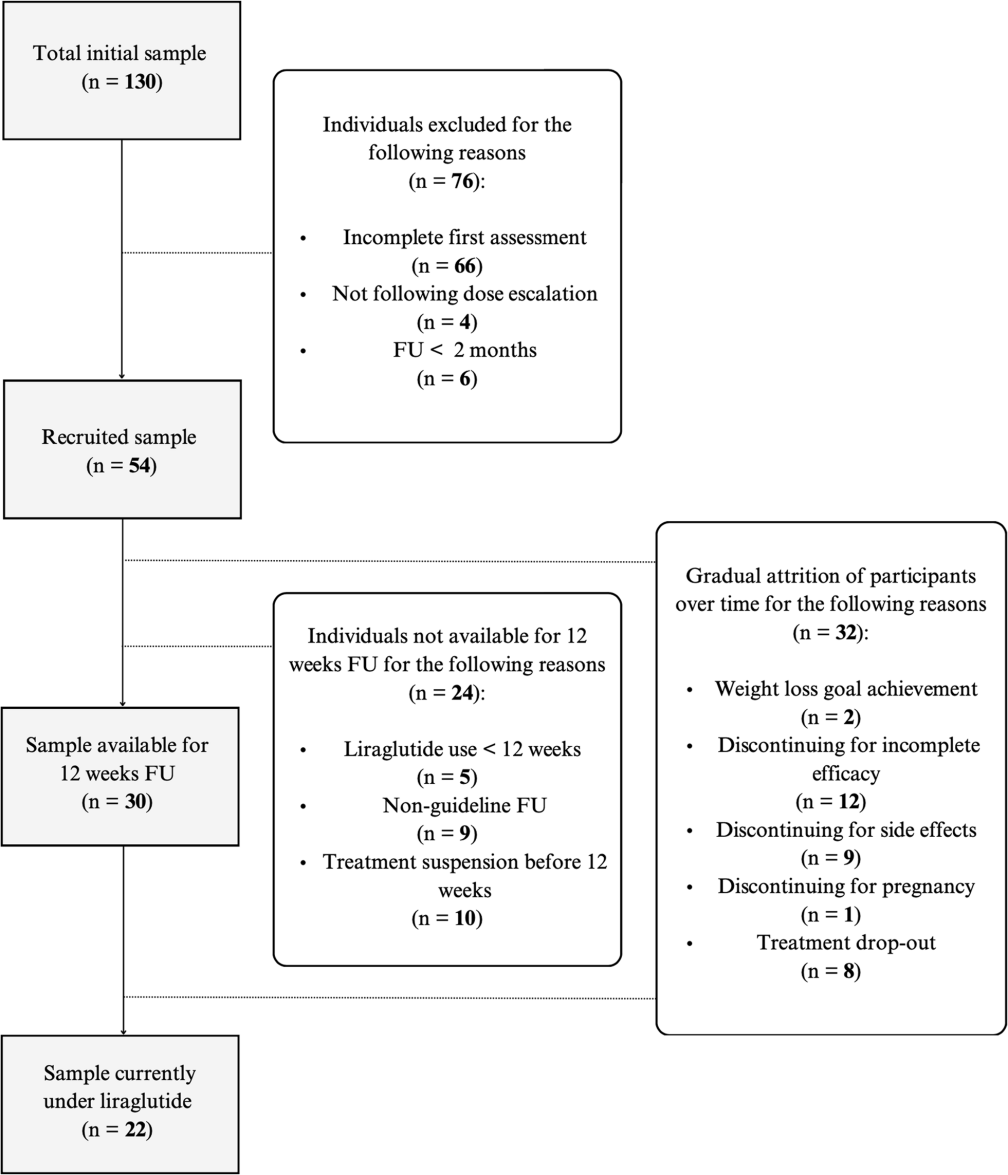
Flowchart: sample selection process and progressive attrition of participants. *FU* follow-up, *n* number of subjects*, < * less than

The flowchart in Fig. 1 offers a visual representation of the sample selection process, illustrating the progressive attrition of participants. Of 130 individuals with obesity on liraglutide therapy, 66 were excluded for not having completed the psychometric assessment, four were excluded for taking liraglutide without following the recommended dosing escalation schedule as described in the drug datasheet and/or as prescribed by the clinician, and six were excluded since they had a follow-up shorter than 2 months. The final sample included 54 subjects, of which the majority (n = 36; 66.7%) were female. The mean age was 48.56 (± 13 SD) years, ranging from 19 to 73 years. The mean BMI was 39.89 (± 6.05 SD) kg/m^2^, ranging from 27.2 kg/m^2^ to 59.5 kg/m^2^. The average weight loss in the overall sample (*n* = 54) was 4.43% (± 5.5 SD); it attained 4.67% (± 5.28 SD) in the female population and 3.95% (± 6.05 SD) in the male population. Thirty patients were available for evaluation of treatment effectiveness at 12 weeks (i.e., the schedule indicated in the European Medicine Agency drug prescription sheet). The subcohort exhibited an average weight loss of 5.3% (± 6.3 SD). More specifically, females showed a 5.1% reduction (± 5.8 SD) in weight, while males demonstrated a 5.7% reduction (± 8 SD).

Over the clinical observation period, 32 out of 54 patients (59.26% of the sample) discontinued the drug, reflecting a gradual attrition of participants throughout the follow-up period. The attrition was due to several factors, each contributing to participant discontinuation. Specifically, two participants (6.25%) discontinued their participation upon achieving their weight loss goals. Twelve individuals (37.5% of the total discontinuations) stopped liraglutide due to perceived incomplete efficacy, and among these, only 5 had reached the maximum dosage of 3 mg. Gastrointestinal side effects (i.e., nausea, emesis, gastralgia) were reported as the primary reason for discontinuation by nine participants (28.13%). An additional eight participants dropped out, i.e., interrupted treatment follow-up (25%). Finally, one participant (3.13%) had to discontinue treatment due to the occurrence of pregnancy. These attrition factors reflect the complexities and challenges encountered during the research process.

### Psychiatric symptoms, psychoactive treatments, and adherence indicators

Key results are summarized in Tables 1 and 2. Regarding anxiety, we found mean STAI values of 44.48 (± 9.18 SD), indicative of mild anxiety. Looking at the distribution, we identified that 35.2% (n = 19) of subjects did not suffer from clinically significant anxiety (STAI score < 40), 37% (*n* = 20) of subjects suffered from mild anxiety (STAI score 40–49), 24.1% (*n* = 13) of subjects suffered from moderate anxiety (STAI score 50–59), and 3.7% (*n* = 2) suffered from severe anxiety (STAI ≥ 60).

**Table 1:** Psychometric scales and adherence indicators

	Minimum	Maximum	Mean value ± SD
STAI score	28	68	44.48 ± 9.18
BDI score	0	37	13.54 ± 8.12
BES score	0	35	15.46 ± 9.59
MD (mg)	0.6	3	2.1 ± 0.71
TD (days)	0	632	177.2 ± 155.2

*BDI* Beck Depression Inventory, *BES* Binge Eating Scale, *MD* Maximum Dosage, *SD* standard deviation, *STAI* State-Trait Anxiety Inventory, *TD* treatment duration

**Table 2:** Psychometric scales graded by severity

	Below threshold	Mild	Moderate	Severe
	N; %	N; %	N; %	N; %
STAI	19; 35.2%	20; 37%	13; 24.1%	2; 3.7%
BDI	17; 31.5%	25; 46.3%	10; 18.5%	2; 3.7%
BES	35; 64.8%	–	10; 18.5%	9; 16.7%

*BDI* Beck Depression Inventory, *BES* Binge Eating Scale, *N* number of subjects, *SD* standard deviation, *STAI* State-Trait Anxiety Inventory; %, percent

Concerning depression, we found mean BDI values of 13.54 (± 8.12 SD), indicative of mild depression. Looking at the distribution, we identified that 31.5% (*n* = 17) of subjects did not suffer from clinically significant depression (BDI score < 10), 46.3% (*n* = 25) of subjects suffered from mild depression (BDI score 10–19), 18.5% (*n* = 10) of subjects suffered from moderate depression (BDI score 20–29), and 3.7% (n = 2) suffered from severe depression (BDI score ≥ 30).

Regarding binge eating, we identified mean BES values of 15.46 (± 9.59 SD), indicative of no significant BE. Indeed, 64.8% (*n* = 35) of the sample had no significant BE (BES score < 17). The remaining 35% (*n* = 29) had significant BE (BES score > 17): 18.5% (*n* = 10) of the sample had moderate levels of BE (BES score 17–26), and 16.7% (*n* = 9) had severe levels of BE (BES score ≥ 26).

Twelve patients were receiving single-drug treatments for antidepressant and/or antianxiety and/or antipsychotic purposes. These medications included three individuals on vortioxetine, three on escitalopram, two on sertraline, one on paroxetine, one on duloxetine, one on aripiprazole, and one on etizolam. Additionally, four patients were undergoing combination psychotropic treatments, with one patient on sodium valproate combined with sertraline and trazodone, another on olanzapine along with trazodone and pregabalin, one on venlafaxine alongside trazodone and pregabalin, and the fourth patient receiving olanzapine in conjunction with fluoxetine. Furthermore, in two cases, antidepressant therapy (one on venlafaxine and one on fluoxetine) commenced concurrently with the initiation of liraglutide. In aggregate, it is noteworthy that approximately one-third of the patients had been prescribed psychotropic treatments while undergoing liraglutide therapy.

MD had a range between 0.6 mg and 3 mg, with a mean value of 2.1 mg (± 0.71 SD). TD had a mean value of 177.2 days (± 155.2 SD), with a minimum of 0 days and a maximum of 632 days.

### Associations between psychiatric symptoms or medications and adherence indicators

**Fig. 2:**
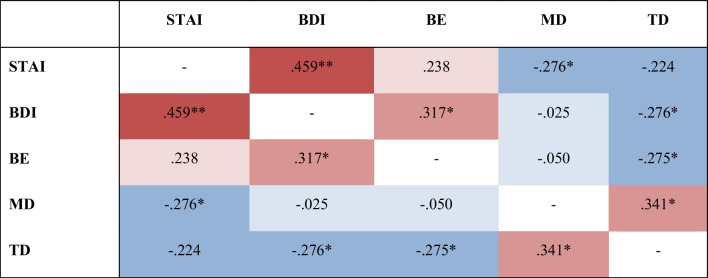
Pearson's correlations Heatmap. Higher correlations are marked with darker shades, lower correlations with lighter shades. *BDI* Beck Depression Inventory, *BES* Binge Eating Scale, *MD* maximum dosage, *STAI* State-Trait Anxiety Inventory, *TD* treatment duration; *p-value < 0.05; **p-value < 0.001

Bivariate correlations analyses are visually depicted in the heatmap shown in Fig. 2. Our analyses demonstrated a negative correlation between STAI and MD (*r* = − 0.276; *p* < 0.05) and a negative correlation between BDI and TD (*r* = − 0.276; *p* < 0.05). Since no significant correlation emerged between BES and adherence indicators, we created the categorical variable binge eating (BE) based on its BES cut-off (BES > 17): we found a negative correlation between a significant BE and TD (*r* = − 0.275; *p* < 0.05).

Linear regression analyses—carried out on variables with significant results in correlation analyses—demonstrated a significant association between STAI and MD (*β* = − 0.021; SE = 0.010; *t* = − 2.068; *p* < 0.05; *F* (1, 52) = 4.279; *R*^2^ = 0.058), BDI and TD (*β* = − 5.271; SE = 2.548; *t* = − 2.069; *p* < 0.05; *F* (1, 52) = 4.280; R^2^ = 0.058), and BE and TD (*β* = − 88.582; SE = 42.930; *t* = − 2.063; *p* < 0.05; *F* (1, 52) = 4.258; *R*^2^ = 0.058).

These results highlight that greater anxiety symptoms can be associated with a lower MD; greater depressive symptoms and a significant BE can be associated with a shorter TD.

Through Student’s *T*-test analysis, we examined the influence of psychotropic treatment on both MD and TD and observed no significant differences between individuals receiving psychotropic treatment and those who were not.

### Relationship between psychiatric symptoms or medications and weight loss

Investigating the relationship between psychiatric symptoms, medication use, and weight loss, we found no significant correlations between psychometric scores and percent weight loss in either the full sample of 54 participants or the subset of patients available for the 12-week treatment evaluation (*n* = 30). Additionally, no significant correlations were observed between psychotropic medication use and percent weight loss in both the full sample and the 12-week evaluation subset.

## Discussion

To the best of our knowledge, this is the second real-world study investigating the relationship between psychiatric symptoms and adherence to liraglutide 3.0 mg as obesity therapy. The other one was also carried out in Italy in a smaller study on 29 patients, which—unlike ours—were previously selected as having established bipolar or major depressive disorder [30]. The attrition of participants in our study was notably high, with 59.26% of the sample discontinuing treatment during the clinical observation period. In our study, various factors contributed to treatment discontinuation, including perceived incomplete treatment efficacy, gastrointestinal side effects, and program drop-out. Only two individuals discontinued treatment upon reaching their weight goals. This attrition rate, far exceeding the 28.1% reported in the SCALE Obesity and Prediabetes clinical trial [18], was also higher than that reported in another other real-world study in Switzerland where psychiatric symptoms were not assessed [16], but comparable to that found in patients with established psychiatric diagnosis [30]. This underscores the challenges faced in real-world clinical settings when using GLP-1 agonists for obesity treatment.

Analysis of psychiatric symptoms, including anxiety, depression, and binge eating, revealed that a significant portion of the sample experienced mild to moderate levels of these symptoms. Around one-third of the patients were receiving single-drug or combination psychotropic treatments alongside liraglutide therapy. In the subset of patients available for the 12-week treatment evaluation, we found a noteworthy inverse correlation between psychiatric symptoms and adherence indicators. Subsequent regression analysis has provided evidence suggesting that heightened anxiety symptomatology might be associated with a reduced MD, while elevated depressive symptomatology and significant BE may correspond to a shorter TD. Nevertheless, we were able to observe that, despite the effect on adherence, neither the presence of psychiatric symptoms nor being on psychoactive therapies led to a significant reduction in overall liraglutide effectiveness on weight loss.

Our results are consistent with previous research indicating suboptimal treatment adherence among individuals with obesity who also exhibit symptoms of anxiety and depression [31]. It is widely recognized that depression and anxiety are frequently linked to reduced compliance and adherence to medication regimens [32–35]. Moreover, there is evidence to suggest that the use of antidepressants may mitigate the weight loss effects of GLP-1 RAs, potentially influencing therapy adherence negatively [36]. Overall, the recurrent co-occurrence of obesity, anxiety-depressive syndromes, and the use of antidepressant therapies often results in a complex interplay, both in clinical presentation and adherence to treatment [37].

In assessing the specific adherence to liraglutide, it is pertinent to account for intrinsic factors associated with the medication. Firstly, it is worth noting that mild and transient side effects, including nausea, constipation, diarrhea, and vomiting, may be variably tolerated, potentially influenced by the presence of anxiety [18, 38]. Secondly, it is essential to consider that medications requiring out-of-pocket expenses, as exemplified by liraglutide 3.0 mg for Italian patients, have been shown to exert a negative influence on adherence [39, 40]. In the course of this audit, the use of psychotropic therapies, predominantly antidepressants, did not appear to have a discernible impact on adherence.

Could increased adherence leading to weight loss—in persons with obesity and psychiatric symptoms—potentially establish a positive feedback loop where physical and mental well-being mutually reinforce each other? Indeed, prior research has demonstrated a bidirectional relationship between obesity and depression [41, 42], inflammation and depression [43, 44], obesity and inflammation [45, 46], and inflammation and anxiety [47]. This body of research has illustrated that states of anxiety and depression, alongside obesity, bring about analogous changes in central nervous system cells, primarily attributed to the heightened activity of glucocorticoids, proinflammatory cytokines, and glutamate [20, 21, 41]. Notably, an excess of glucocorticoids can give rise to compromised insulin function and glucose metabolism, limit the availability of energy necessary for optimal neuronal function, and consequently disrupt synaptic plasticity [20, 21, 41].

Despite the evidence from in vitro and animal studies, there is still much uncertainty about whether weight loss—regardless of the methods used—can improve mental health. Lifestyle modification can provide greater reductions in symptoms of depression than with alternative weight loss interventions, including dietary counseling and exercise-alone, while obesity pharmacotherapy yielded contradictory results depending on the drug used [25]. Anti-hyperglycemic agents, such as metformin and GLP-1 RAs, have consistently shown to exert anti-depressive properties [21]. Indeed, there is wide evidence that the use of GLP-1 RAs in T2DM patients is associated with a lower incidence of depression and anxiety compared to controls treated with different therapies [23, 48, 49]. In addition, recent studies evaluating the gut microbiota have suggested that one of the roles of GLP-1 RAs in treating anxiety is related to improved glucoregulation, leading to reduced proinflammatory cytokines and increased neuroprotection [50]. Conversely, a warning has been recently issued about a possible association between liraglutide use and increased suicidal behavior and thoughts in Iceland, which prompted a retrospective review of GLP-1 RAs by the European Medicines Agency [51]. Future prospective studies on GLP-1 RAs and psychiatric symptoms are therefore warranted, possibly also investigating putative mechanisms directly affecting the CNS.

It is worth noting that within the arsenal of treatments for obesity, there is another medication known as naltrexone/bupropion. This compound represents a fixed-dose synergistic combination of two molecules originally employed in the treatment of opioid/alcohol use disorders and depression [52]. Notably, it has demonstrated effectiveness in curbing appetite and boosting energy expenditure, which aids individuals in adhering to a calorie-controlled diet and ultimately shedding excess body weight. According to the guidelines provided by the Canadian healthcare system, naltrexone/bupropion is recommended as the primary pharmacological option for individuals with a body mass index (BMI) of 30 kg/m^2^ or greater, or for those with a BMI of 27 kg/m^2^ or greater accompanied by obesity-related comorbidities, particularly when patients exhibit symptoms such as food cravings or depression [53].

Therefore, there are numerous reasons not only to counteract clinical inertia by providing pharmacological obesity treatment to patients with psychiatric symptoms, but also to direct therapy toward pathophysiological pathways shared between physical and mental health. It becomes evident that the care afforded to one facet inherently extends to the betterment of the other.

### Strength and limits

Strengths of this study include the real-life setting in a public outpatient obesity clinic, therefore less prone to selection bias than randomized controlled trials—which tend to select those patients with higher adherence [54, 55]. This setting was also more representative of the problems clinicians can face while prescribing a drug that is not covered by the National Health Service—unlike most medications for clinically relevant diseases. Furthermore, this study is based on the routine collection of psychiatric symptoms in the context of the first visit to the Unit of Clinical Nutrition and Metabolism, performing in the internal medicine setting, a type of assessment that is generally reserved for the psychiatric setting.

The present study is subject to several methodological limitations that must be acknowledged. Firstly, the sample size employed in this research consisted of only 54 participants. This limited sample size may have hindered the generalizability of the findings and could have led to an increased susceptibility to sampling bias. Additionally, the study's relatively short duration, with an average TD of 177.2 days (± 155.2 days), may have limited our ability to conduct a thorough assessment of long-term effects or trends. Furthermore, the presence of substantial missing data at both baseline and follow-up assessments is a notable concern, potentially introducing biases and reducing the robustness of the statistical analyses. These missing data may have resulted from various factors, including participant attrition or data collection challenges, which were exacerbated by logistical difficulties related to the Covid-19 pandemic. In addition, this clinical audit involved retrospective observations of subjects, and only half of the sample successfully completed or returned the psychodiagnostic scales, primarily due to the pandemic-related challenges. Moreover, there were limitations in tracking and documenting the reasons for treatment discontinuation, which impeded the possibility of conducting more specific statistical analyses on the issue of side effects. Lastly, the use of self-report questionnaires for assessing psychiatric symptoms, associated with potentially high false positive and negative rates, combined with variations in the timing of these assessments before the initiation of the weight loss drug, may have imposed limitations on the data's validity. Therefore, it is imperative to interpret the results with caution, considering these multiple limitations when assessing the implications of the study.

### Conclusions

The study reveals an initial discontinuation rate of approximately 60% within a real-life clinical setting. While the study limitations do not allow a definite conclusion on whether psychiatric symptoms might play a role in diminishing adherence to liraglutide 3.0 mg therapy in such practical contexts, it is essential to emphasize that these symptoms do not seem to impede the drug’s capacity to promote weight loss. This finding underscores the potential advantages of liraglutide 3.0 mg therapy for individuals contending with obesity while simultaneously managing mental health challenges.

Advancing our comprehension of this relationship between psychiatric symptoms, including depression, anxiety, and binge eating, and adherence to Liraglutide 3.0 therapy may facilitate the identification of tailored interventions for our patient cohort. An integrative approach to the management of such individuals has the potential to ameliorate psychiatric symptoms and enhance therapeutic adherence, ultimately contributing to the broader improvement of their overall health.

## Data Availability

The dataset analyzed during the current study is available from the corresponding author upon request.

## References

[1] Sabate E (2003). Adherence to long term therapies: evidence for action. Geneva: World Health Organization. https://apps.who.int/iris/handle/10665/42682

[2] Haslam DW, James WP (2005). Obesity. Lancet.

[3] Smith KB, Smith MS (2016). Obesity statistics. Prim Care.

[4] Burgess E, Hassmén P, Pumpa KL (2017). Determinants of adherence to lifestyle intervention in adults with obesity: a systematic review. Clin Obes.

[5] Kim H, Kim MS, Lee JE, Kim JW, Lee CH, Yoon IY, Rhee CS (2013). Treatment outcomes and compliance according to obesity in patients with obstructive sleep apnea. Eur Arch Otorhinolaryngol.

[6] Golden A (2017). Current pharmacotherapies for obesity: a practical perspective. J Am Assoc Nurse Pract.

[7] Guerrero-Hreins E, Goldstone AP, Brown RM, Sumithran P (2021). The therapeutic potential of GLP-1 analogues for stress-related eating and role of GLP-1 in stress, emotion and mood: a review. Prog Neuropsychopharmacol Biol Psychiatry.

[8] Tsapas A, Avgerinos I, Karagiannis T, Malandris K, Manolopoulos A, Andreadis P, Liakos A, Matthews DR, Bekiari E (2020). Comparative effectiveness of glucose-lowering drugs for type 2 diabetes: a systematic review and network meta-analysis. Ann Intern Med.

[9] Shyangdan DS, Royle P, Clar C, Sharma P, Waugh N, Snaith A (2011). Glucagon-like peptide analogues for type 2 diabetes mellitus. Cochrane Database Syst Rev.

[10] Amori RE, Lau J, Pittas AG (2007). Efficacy and safety of incretin therapy in type 2 diabetes: systematic review and meta-analysis. JAMA.

[11] Nauck MA, Quast DR, Wefers J, Meier JJ (2021). GLP-1 receptor agonists in the treatment of type 2 diabetes—state-of-the-art. Mol Metab.

[12] Rowlands J, Heng J, Newsholme P, Carlessi R (2018). Pleiotropic effects of GLP-1 and analogs on cell signaling, metabolism, and function. Front Endocrinol (Lausanne).

[13] Drucker DJ (2022). GLP-1 physiology informs the pharmacotherapy of obesity. Mol Metab.

[14] Iqbal J, Wu HX, Hu N, Zhou YH, Li L, Xiao F, Wang T, Jiang HL, Xu SN, Huang BL, Zhou HD (2022). Effect of glucagon-like peptide-1 receptor agonists on body weight in adults with obesity without diabetes mellitus-a systematic review and meta-analysis of randomized control trials. Obes Rev.

[15] Ryan PM, Seltzer S, Hayward NE, Rodriguez DA, Sless RT, Hawkes CP (2021). Safety and efficacy of glucagon-like peptide-1 receptor agonists in children and adolescents with obesity: a meta-analysis. J Pediatr.

[16] Haase CL, Serratore Achenbach MG, Lucrezi G, Jeswani N, Maurer S, Egermann U (2021). Use of liraglutide 3.0 mg for weight management in a real-world setting in Switzerland. Obes Facts.

[17] FDA, 2020. Saxenda FDA approval history. https://www.drugs.com/history/saxenda.html

[18] Pi-Sunyer X, Astrup A, Fujioka K, Greenway F, Halpern A, Krempf M, Lau DC, le Roux CW, Violante Ortiz R, Jensen CB, Wilding JP (2015). SCALE Obesity and Prediabetes NN8022–1839 Study Group. A Randomized, Controlled Trial of 30 mg of Liraglutide in Weight Management. N Engl J Med.

[19] O'Neil PM, Birkenfeld AL, McGowan B, Mosenzon O, Pedersen SD, Wharton S, Carson CG, Jepsen CH, Kabisch M, Wilding JPH (2018). Efficacy and safety of semaglutide compared with liraglutide and placebo for weight loss in patients with obesity: a randomised, double-blind, placebo and active controlled, dose-ranging, phase 2 trial. Lancet.

[20] Detka J, Głombik K (2021). Insights into a possible role of glucagon-like peptide-1 receptor agonists in the treatment of depression. Pharmacol Rep.

[21] Essmat N, Soliman E, Mahmoud MF, Mahmoud AAA (2020). Antidepressant activity of anti-hyperglycemic agents in experimental models: a review. Diabetes Metab.

[22] Turan I, Sayan Ozacmak H, Ozacmak VH, Ergenc M, Bayraktaroğlu T (2021). The effects of glucagon-like peptide 1 receptor agonist (exenatide) on memory impairment, and anxiety- and depression-like behavior induced by REM sleep deprivation. Brain Res Bull.

[23] Tsai WH, Sung FC, Chiu LT, Shih YH, Tsai MC, Wu SI (2022). Decreased risk of anxiety in diabetic patients receiving glucagon-like peptide-1 receptor agonist: a nationwide. Population-based cohort study. Front Pharmacol.

[24] Apperley LJ, Gait L, Erlandson-Parry K, Laing P, Senniappan S (2021). Liraglutide combined with intense lifestyle modification in the management of obesity in adolescents. J Pediatr Endocrinol Metab.

[25] Fabricatore AN, Wadden TA, Higginbotham AJ, Faulconbridge LF, Nguyen AM, Heymsfield SB, Faith MS (2011). Intentional weight loss and changes in symptoms of depression: a systematic review and meta-analysis. Int J Obes (Lond).

[26] Spielberger CD, Gorsuch RL, Lushene R, Vagg PR, Jacobs GA (1983). Manual for the state-trait anxiety inventory.

[27] Beck AT, Ward CH, Mendelson M, Mock J, Erbaugh J (1961). An inventory for measuring depression. Arch Gen Psychiatry.

[28] Gormally J, Black S, Daston S, Rardin D (1982). The assessment of binge eating severity among obese persons. Addict Behav.

[29] Halseth A, Shan K, Gilder K, Malone M, Acevedo L, Fujioka K (2018). Quality of life, binge eating and sexual function in participants treated for obesity with sustained release naltrexone/bupropion. Obes Sci Pract.

[30] Cuomo A, Bolognesi S, Goracci A, Ciuoli C, Beccarini Crescenzi B, Maina G, Rosso G, Facchi E, Maccora C, Giordano N, Verdino V, Fagiolini A (2019). Feasibility, adherence and efficacy of liraglutide treatment in a sample of individuals with mood disorders and obesity. Front Psychiatry.

[31] Hoffmann K, Kopciuch D, Michalak M, Bryl W, Kus K, Marzec K, Raakow J, Pross M, Berghaus R, Nowakowska E, Kostrzewska M, Zaprutko T, Ratajczak P, Paczkowska A (2022). Adherence of obese patients from Poland and Germany and its impact on the effectiveness of morbid obesity treatment. Nutrients.

[32] Evans L, Spelman M (1983). The problem of non-compliance with drug therapy. Drugs.

[33] Gat A, Mathes T (2019). Medication adherence influencing factors-an (updated) overview of systematic reviews. Syst Rev.

[34] Rossom RC, Shortreed S, Coleman KJ, Beck A, Waitzfelder BE, Stewart C, Ahmedani BK, Zeber JE, Simon GE (2016). Antidepressant adherence across diverse populations and healthcare settings. Depress Anxiety.

[35] Sirey JA, Bruce ML, Alexopoulos GS, Perlick DA, Friedman SJ, Meyers BS (2001). Stigma as a barrier to recovery: perceived stigma and patient-rated severity of illness as predictors of antidepressant drug adherence. Psychiatr Serv.

[36] Durell N, Franks R, Coon S, Cowart K, Carris NW (2022). Effect of antidepressants on glucagon-like peptide-1 receptor agonist-related weight loss. J Pharm Technol.

[37] Fulton S, Décarie-Spain L, Fioramonti X, Guiard B, Nakajima S (2021). The menace of obesity to depression and anxiety prevalence. Trends Endocrinol Metab.

[38] Zhang P, Liu Y, Ren Y, Bai J, Zhang G, Cui Y (2019). The efficacy and safety of liraglutide in the obese, non-diabetic individuals: a systematic review and meta-analysis. Afr Health Sci.

[39] Eaddy MT, Cook CL, O’Day K, Burch SP, Cantrell CR (2012). How patient cost-sharing trends affect adherence and outcomes: a literature review. P T.

[40] Sinnott SJ, Buckley C, O’Riordan D, Bradley C, Whelton H (2013). The effect of copayments for prescriptions on adherence to prescription medicines in publicly insured populations; a systematic review and meta-analysis. PLoS ONE.

[41] Milaneschi Y, Simmons WK, van Rossum EFC, Penninx BW (2019). Depression and obesity: evidence of shared biological mechanisms. Mol Psychiatry.

[42] Jantaratnotai N, Mosikanon K, Lee Y, McIntyre RS (2017). The interface of depression and obesity. Obes Res Clin Pract.

[43] Beurel E, Toups M, Nemeroff CB (2020). The bidirectional relationship of depression and inflammation: double trouble. Neuron.

[44] Orsolini L, Pompili S, Tempia Valenta S, Salvi V, Volpe U (2022). C-reactive protein as a biomarker for major depressive disorder?. Int J Mol Sci.

[45] Kawai T, Autieri MV, Scalia R (2021). Adipose tissue inflammation and metabolic dysfunction in obesity. Am J Physiol Cell Physiol.

[46] Cox AJ, West NP, Cripps AW (2015). Obesity, inflammation, and the gut microbiota. Lancet Diabetes Endocrinol.

[47] Michopoulos V, Powers A, Gillespie CF, Ressler KJ, Jovanovic T (2017). Inflammation in fear- and anxiety-based disorders: PTSD, GAD, and beyond. Neuropsychopharmacology.

[48] Grant P, Lipscomb D, Quin J (2011). Psychological and quality of life changes in patients using GLP-1 analogues. J Diabetes Complications.

[49] Moulton CD, Pickup JC, Amiel SA, Winkley K, Ismail K (2016). Investigating incretin-based therapies as a novel treatment for depression in type 2 diabetes: findings from the South London Diabetes (SOUL-D) Study. Prim Care Diabetes.

[50] Lach G, Schellekens H, Dinan TG, Cryan JF (2018). Anxiety, Depression, and the Microbiome: A Role for Gut Peptides. Neurotherapeutics.

[51] European Medication Agency, EMA statement on ongoing review of GLP-1 receptor agonists. https://www.ema.europa.eu/en/news/ema-statement-ongoing-review-glp-1-receptor-agonist. Accessed 11 Jul 2023.

[52] EMA, 2015. Mysimba EMA Approval History. https://www.ema.europa.eu/en/medicines/human/EPAR/mysimba

[53] Pedersen SD, Manjoo P, Wharton S. Canadian Adult Obesity Clinical Practice Guidelines: Pharmacotherapy in Obesity Management. Available from:https://obesitycanada.ca/guidelines/pharmacotherapy. Accessed 30 Nov 2022.

[54] Sanson-Fisher RW, Bonevski B, Green LW, D'Este C (2007). Limitations of the randomized controlled trial in evaluating population-based health interventions. Am J Prev Med.

[55] Zeilstra D, Younes JA, Brummer RJ, Kleerebezem M (2018). Perspective: fundamental limitations of the randomized controlled trial method in nutritional research: the example of probiotics. Adv Nutr.

